# The PI3Kδ Inhibitor Idelalisib Diminishes Platelet Function and Shows Antithrombotic Potential

**DOI:** 10.3390/ijms22073304

**Published:** 2021-03-24

**Authors:** María N. Barrachina, Irene Izquierdo, Lidia Hermida-Nogueira, Luis A. Morán, Amparo Pérez, Ana B. Arroyo, Nuria García-Barberá, Rocío González-Conejero, Sara Troitiño, Johannes A. Eble, José Rivera, Constantino Martínez, María I. Loza, Eduardo Domínguez, Ángel García

**Affiliations:** 1 Platelet Proteomics Group, Center for Research in Molecular Medicine and Chronic Diseases, Universidade Santiago de Compostela and Instituto de Investigación Sanitaria de Santiago, 15706 Santiago de Compostela, Spain; Maria.Barrachina@childrens.harvard.edu (M.N.B.); irene.izquierdo.b@gmail.com (I.I.); lidia.nogueira@usc.es (L.H.-N.); luisarturo.moran@usc.es (L.A.M.); sara.troitino.cora@rai.usc.es (S.T.); 2 Pharmacology Applied to Drug Discovery Group, Centro Singular de Investigación en Medicina Molecular y Enfermedades Crónicas, Universidade Santiago de Compostela, 15705 Santiago de Compostela, Spain; amparo.perez@usc.es (A.P.); mabel.loza@usc.es (M.I.L.); eduardo.dominguez@usc.es (E.D.); 3 Grupo Biofarma, Instituto de Investigación Sanitaria de Santiago, 15706 Santiago de Compostela, Spain; 4 Servicio de Hematología y Oncología Médica, Hospital Universitario Morales Meseguer, Centro Regional de Hemodonación, Universidad de Murcia, IMIB-Arrixaca, CIBERER-U765, 30003 Murcia, Spain; anabelen.arroyo@um.es (A.B.A.); nurgarbar@gmail.com (N.G.-B.); rocio.gonzalez@carm.es (R.G.-C.); jose.rivera@carm.es (J.R.); constant@um.es (C.M.); 5 Institute of Physiological Chemistry and Pathobiochemistry, University of Münster, 48149 Münster, Germany; johannes.eble@uni-muenster.de

**Keywords:** platelets, Idelalisib, PI3K inhibitors

## Abstract

Background: Clinical management of ischemic events and prevention of vascular disease is based on antiplatelet drugs. Given the relevance of phosphatidylinositol-4,5-bisphosphate 3-kinase (PI3K) as a candidate target in thrombosis, the main goal of the present study was to identify novel antiplatelet agents within the existing inhibitors blocking PI3K isoforms. Methods: We performed a biological evaluation of the pharmacological activity of PI3K inhibitors in platelets. The effect of the inhibitors was evaluated in intracellular calcium release and platelet functional assays, the latter including aggregation, adhesion, and viability assays. The in vivo drug antithrombotic potential was assessed in mice undergoing chemically induced arterial occlusion, and the associated hemorrhagic risk evaluated by measuring the tail bleeding time. Results: We show that PI3K Class IA inhibitors potently block calcium mobilization in human platelets. The PI3K p110δ inhibitor Idelalisib inhibits platelet aggregation mediated by ITAM receptors GPVI and CLEC-2, preferentially by the former. Moreover, Idelalisib also inhibits platelet adhesion and aggregation under shear and adhesion to collagen. Interestingly, an antithrombotic effect was observed in mice treated with Idelalisib, with mild bleeding effects at high doses of the drug. Conclusion: Idelalisib may have antiplatelet effects with minor bleeding effects, which provides a rationale to evaluate its antithrombotic efficacy in humans.

## 1. Introduction

Cardiovascular diseases (CVD), specially ischemic heart disease and stroke, are one of the main causes of death in the world [1]. Platelets contribute to hemostasis by their activation, aggregation, and adherence into a plug in cases of vascular trauma; on the other hand, uncontrolled platelet activation may lead to complete vessel occlusion (thrombosis), and thereby life-threatening ischemia in heart, brain, or other organs [2].

Current antiplatelet drugs used for the prevention and treatment of thrombosis target four major classes of proteins, including cyclooxygenase 1 (aspirin), P2Y12 (e.g., clopidogrel, prasugrel, and ticagrelor), integrin αIIbβ3 (e.g., abciximab), and protease-activated receptor 1 (PAR1) (vorapaxar) [3]. A fundamental challenge in antithrombotic drug discovery is to develop antiplatelet agents capable of preventing thrombus while having a minor impact on bleeding. 

Intracellular platelet signaling pathways, such as those mediated by phosphatidylinositol-4,5-bisphosphate 3-kinase (PI3K), are target candidates for anti-thrombosis drug development. Indeed, PI3K is required for platelet activation and adhesion as well as thrombus formation [4,5]. PI3K Class I isoforms are subdivided into class IA (p110α, p110β, and p110δ) and class IB (p110γ). These enzymes contribute to many cellular processes, such as proliferation and survival and metabolism control through their activation by cell surface receptors, inducing the synthesis of PIP3 [6]. To date, efforts to target PI3Ks in thrombosis have been focused on PI3Kβ, which is blocked by the selective PI3K inhibitor TGX-221, and by the improved structural analogue AZD6482 and other inhibitors, which have shown antiplatelet activity [7,8,9].

Due to the potential of PI3K inhibition as an antithrombotic therapy, the primary goal of the present study was the identification of novel antiplatelet drugs able to target different isoforms of PI3K. This was done by performing a pharmacological evaluation of PI3K inhibitors. To address this objective, we developed a phenotypic assay to measure the intracellular calcium mobilization in real time upon drug-induced platelet activation. Selected PI3K inhibitors were further evaluated in in vitro human platelet functional assays, including aggregation and adhesion assays as well as viability assays. Finally, the in vivo antithrombotic efficacy of the selected drug was assessed in a murine model of chemically-induced arterial occlusion and the hemorrhagic risk evaluated by measuring the tail bleeding time.

## 2. Results

### 2.1. Prioritization of PI3K-Related Inhibitors as Antiplatelet Agents by Using an Intracellular Ca^2+^ Mobilization Phenotypic Assay

**Figure 1:**
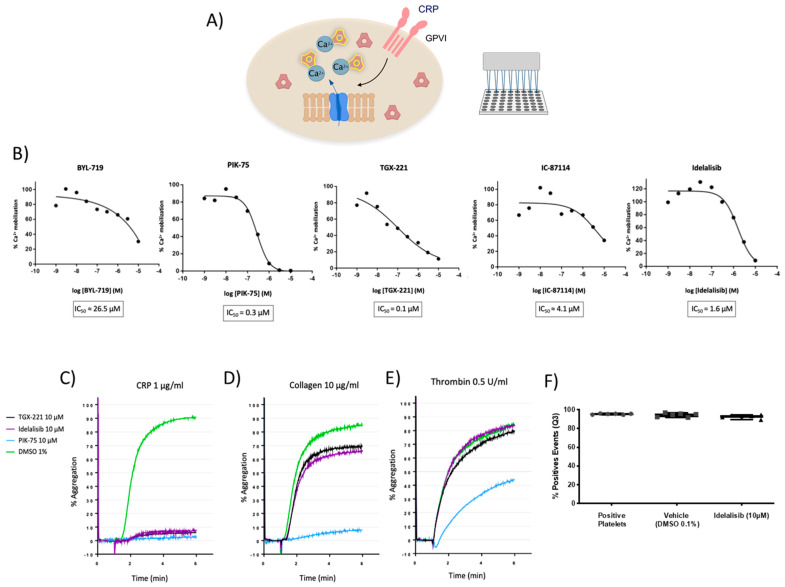
Effect of PI3K inhibitors on platelet activation based on intracellular calcium mobilization and aggregation assays. (**A**) Schematic representation of the intracellular calcium mobilization assay. For detailed information please see the Methods section. (**B**) TGX-221, Idelalisib, and PIK-75 block intracellular calcium release mediated by Collagen-Related Peptide (CRP)-induced GPVI-activation with micromolar potency, whereas IC-87114 and BYL-719 have a weaker effect. (**C**–**E**) Representative aggregation curves of platelets treated with TGX-221, and Idelalisib, and PIK-75 from two independent experiments. Inhibitors completely block CRP-mediated platelet aggregation with a less pronounced effect inhibiting collagen-mediated aggregation and no effect on thrombin-mediated aggregation. In the case of PIK-75, it blocks, totally or moderately, platelet aggregation induced by either CRP, collagen, or thrombin. (**F**) Calcein-AM-based viability assay demonstrating platelets are viable following 10 µM Idelalisib treatments.

Given the relevance of PI3K in GPVI-mediated platelet activation, we evaluated the effect of five PI3K Class IA inhibitors on intracellular calcium mobilization following platelet activation with the GPVI-specific agonist collagen-related peptide (CRP) (Figure 1A). The inhibitors were the PI3Kα inhibitors BYL-719 and PIK-75, the PI3Kβ inhibitor TGX-221, and the PI3Kδ inhibitors IC-87114 and Idelalisib (also known as Cal-101).

All these compounds inhibited calcium mobilization induced by CRP (Figure 1B). Inhibitors of the three PI3K isoforms, particularly TGX-221, PIK-75, and Idelalisib, were found to block intracellular calcium release with micromolar potency, being their IC_50_ 0.1, 0.3, and 1.6 µM, respectively (Figure 1B). 

### 2.2. GPVI-Mediated Platelet Aggregation Is Preferentially Blocked through p110β and p110δ Inhibition with No Toxic Effects

To examine the role and selectivity of the above hits on platelet activation, we evaluated the effect of pretreating healthy donor washed platelets with 10 µM of TGX-221, Idelalisib, or PIK-75 inhibitors, or DMSO as a vehicle, on agonist-induced platelet aggregation responses. Several agonists were used to activate platelet receptors, such as GPVI (CRP and collagen), α2β1 (collagen), and PAR1/PAR4 (thrombin).

As shown in Figure 1C, PI3K inhibitors TGX-221, Idelalisib, and PIK-75 completely blocked CRP-mediated platelet aggregation at concentrations of 10 µM. In response to collagen, TGX-221 and Idelalisib showed a similar inhibition profile, decreasing aggregation by 20% compared to baseline samples (Figure 1D). However, when washed platelets were stimulated by thrombin in presence of TGX-221 and Idelalisib, aggregation was not affected. On the other hand, the PI3Kα inhibitor PIK-75 totally blocked platelet aggregation induced by either CRP, and collagen, and partly that of thrombin (Figure 1C–E).

Taken together, these results showed that TGX-221 and Idelalisib inhibit platelet aggregation mediated by GPVI activation with a potential preference for this pathway. On the contrary, PIK-75 inhibitor did not show any selectivity among different platelet signaling pathways since it fully blocked both CRP and collagen-induced platelet aggregation and partially blocked thrombin-induced aggregation.

The p110µµ inhibitor TGX-221, which also targets PI3K catalytic subunit δ, was already reported as a potential platelet inhibitor in thrombosis [9]. We examined the putative pharmacological activity of Idelalisib in platelet activation since this drug was not previously considered as an antiplatelet agent and shows an adequate antiplatelet profile. In order to assess whether the observed effects of Idelalisib might be due to toxicity, the viability of human washed platelets treated with the drug, as compared to DMSO, was assessed by flow cytometry with Calcein-AM. As shown in Figure 1F, untreated washed platelets (>90%) were found to be viable and both DMSO and 10 µM Idelalisib treatments have negligible effect on platelet viability.

### 2.3. Idelalisib Inhibits Platelet Aggregation and Adhesion by Favourably Blocking ITAM Signalling Pathways

To further investigate the main activation pathways mediating the inhibition of platelet aggregation by Idelalisib, we studied aggregation in both platelet-rich plasma (PRP) and washed platelets in response to different agonists.

**Figure 2:**
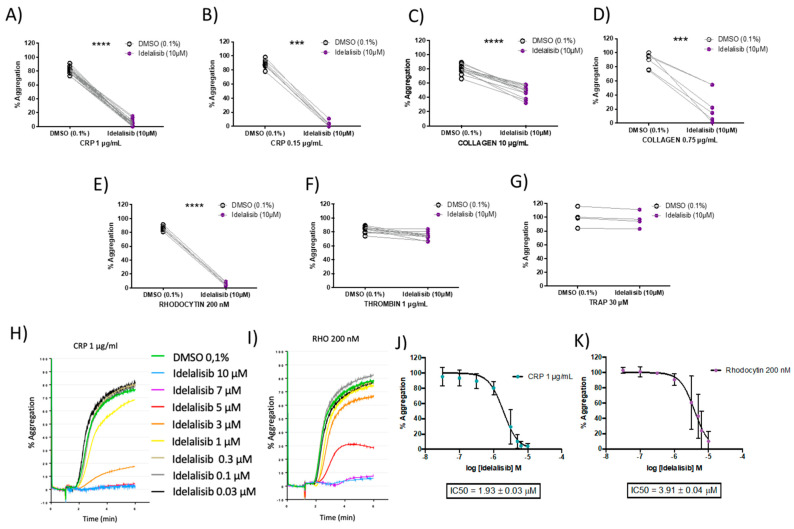
Idelalisib inhibits GPVI-and CLEC-2-mediated platelet aggregation in a dose-response manner. Aggregation profiles upon platelet activation, in presence and absence of Idelalisib, with the following agonists at the indicated doses: (**A**) CRP (washed platelets); (**B**) CRP (platelet-rich-plasma (PRP)); (**C**) collagen (washed platelets); (**D**) collagen (PRP); (**E**) Rhodocytin (washed platelets); (**F**) thrombin (washed platelets); (**G**) thrombin receptor activating peptide (TRAP) (PRP). Effect on aggregation percentage was evaluated in *N* = 22 (CRP, 1 μg/mL), *N* = 13 (Collagen, 5 μg/mL), *N* = 6 (Rhodocytin, 200 nM), and *N* = 11 (Thrombin, 0.1 U/mL), in washed platelets; and *N* = 7 (CRP, 0.15 μg/mL), *N* = 7 (Collagen, 0.75 μg/mL), and *N* = 4 (TRAP, 30 μM) in PRP. H-K) Dose-response efficacy of Idelalisib at inhibiting platelet aggregation following platelet activation in washed platelets with both CRP and Rhodocytin, leading to the determination of IC_50_ values. *** *p* < 0.001; **** *p* < 0.0001. (**H**,**I**) show representative aggregation curves, while (**J**,**K**) plots represent for each concentration used the average of 6 independent measurements from 6 different donors and the error bars are the standard error of the mean.

Idelalisib (10 µM) blocked CRP-XL-induced platelet aggregation, compared with vehicle control, in both PRP and washed platelets from a cohort of human blood donors (Figure 2A,B). The drug also inhibited collagen-induced platelet aggregation (washed platelets and PRP), but to a lesser extent (Figure 2C,D). Total aggregation blockade was also seen in washed platelets stimulated with the CLEC-2 agonist rhodocytin (Figure 2E). On the other hand, Idelalisib showed no inhibition on platelet aggregation when platelets were stimulated with PAR1/PAR4 agonists (thrombin for washed platelets and TRAP for PRP, respectively) (Figure 2F,G).

We then evaluated the effect of a range of different concentrations of Idelalisib (0.03–10 µM) in both CRP- and rhodocytin-induced platelet aggregation responses. Notably, the inhibitory effects of Idelalisib on ITAM-mediated signalling pathways were dose-dependent (Figure 2H,I), with an IC_50_ for CRP-induced aggregation of 1.9 μM (Figure 2J), and 3.9 μM for rhodocytin-induced aggregation in washed platelets (Figure 2K). Thus, Idelalisib shows a similar inhibition profile of platelet activation mediated by GPVI and CLEC-2 receptors.

**Figure 3:**
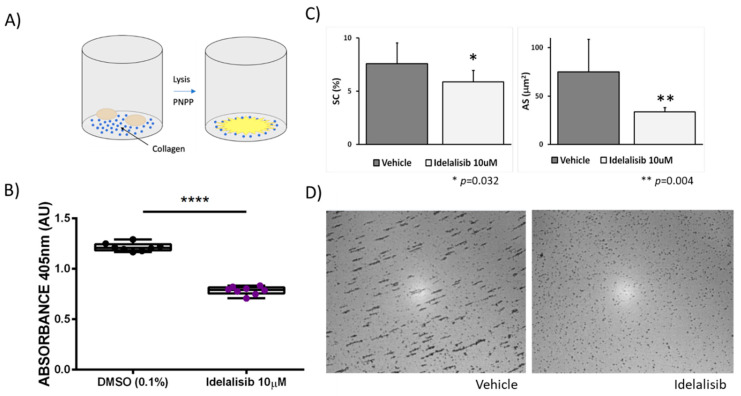
Idelalisib significantly inhibits platelet adhesion to collagen and impairs platelet adhesion and aggregation in the Impact-R test. (**A**) Schematic representation of the adhesion assay. For further information please see the Methods section. (**B**) Inhibition effect of Idelalisib on platelet adhesion to collagen. **** *p* < 0.0001. (**C**) Impact-R test: Plots show the mean values plus standard deviation of the plate surface covered by adhered platelets (%SC) and the average size of the platelet aggregates (AS μm^2^), obtained in blood samples under shear stress, assayed in duplicates, from five different healthy individuals, comparing vehicle samples and samples treated with 10 μM Idelalisib. (**D**) Representative images of the Impact-R evaluation test of untreated blood (vehicle) or blood incubated with 10 μM Idelalisib.

In a separate series of experiments, we examined the effect of p110δ PI3K inhibition on platelet adhesion to type I collagen under static conditions by measuring the absorbance of the enzymatic reaction between p-nitrophenyl phosphate and acid phosphatase (Figure 3A). Vehicle-treated platelets (0.1% DMSO) adhered to collagen, as opposed to platelets treated with Idelalisib (10 µM), whose adhesion is significantly altered (Figure 3B). Moreover, we evaluated the effect of the drug in platelet adhesion and aggregation in the Impact-R test. As show in Figure 3C,D, we found that blood treatment with Idelalisib (10 µM) has a mild effect on the extent of platelet adhesion under shear in the Impact-R test (SC: 7.6 ± 1.9 vs. 5.9 ± 1.1 µm^2^, *p* = 0.032). Moreover, the drug caused a major reduction in platelet aggregation in this test (AS:74.9 ± 33.7 vs. 33.8 ± 4.6 µm^2^, *p* = 0.004). These results further support the concept that Idelalisib may have a role in preventing platelet mediated thrombosis.

### 2.4. Idelalisib Reduces Thrombus Formation with Minor Bleeding Effects in Mice

To investigate more in detail the potential of Idelalisib as antithrombotic, in vivo tail bleeding and a ferric chloride-induced arterial thrombosis assays were performed in a murine model. Mice treated with Idelalisib (20 mg/kg) showed a plasma concentration of 5.1 ± 2.0 µM after 1 h (Supplementary Figure S1).

**Figure 4:**
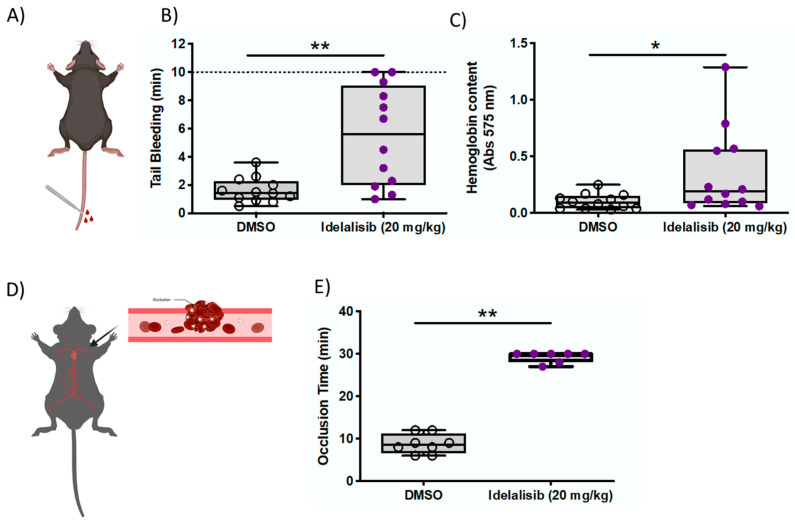
Idelalisib reduces thrombus formation with minor bleeding effects in mice. (**A**) Picture representing the tail bleeding assay. (**B**) The bleeding time was higher in the treated animals although values were always below 10 min. (**C**) The hemoglobin content of blood collected during bleeding was higher in the treated animals. (**D**) Picture representing the FeCl_3_-induced arterial thrombosis model. (**E**) Idelalisib-treated mice display significantly longer occlusion times than control mice. * *p* < 0.05; ** *p* <0.01.

In the tail bleeding assay in mice (Figure 4A), Idelalisib caused a 3.5-fold increase in bleeding time (5.5 ± 3.5 min vs. 1.63 ± 0.88 min of vehicle) (Figure 4B). Accordingly, the hemoglobin content of blood collected during bleeding in Idelalisib-treated mice was significantly higher than in untreated animals (Figure 4C).

Importantly, 9 out of 11 (80%) mice treated with Idelalisib showed bleeding times <10 min. By comparison, 66% of mice treated with ASA or clopidogrel, established antiplatelet therapies, bled over 10 min (Supplementary Figure S2).

FeCl3-induced arterial thrombosis was undertaken to further assess the antithrombotic potential of Idelalisib (Figure 4D). Interestingly, drug-treated mice (20 mg/kg) displayed significantly longer occlusion times than control mice, indicating significant protection against thrombosis in this experimental model (Figure 4E). 

## 3. Discussion

The core findings from the present study are: (i) PI3K Class IA inhibitors potently block calcium mobilization in human platelets; (ii) the drug Idelalisib inhibits platelet aggregation mediated by ITAM receptors GPVI and CLEC-2, without eliciting toxicity; (iii) platelet adhesion to collagen is also inhibited, as well as platelet adhesion and aggregation under shear (Impact-R test with whole blood), in samples treated with Idelalisib; and (iv) a potent antithrombotic and a mild bleeding delay effect was observed in mice treated with Idelalisib.

Antiplatelet therapy has improved during the last decade to significantly enhance the antiplatelet efficacy, while reducing the side effects such as bleeding risk or thrombocytopenia [10]. One such promising target is the PI3K family. Indeed, several studies have demonstrated the role of PI3K regulating different aspects of platelet activation and thrombus formation, including cytoskeletal rearrangements associated with spreading, and the PLCγ2 activation through some of the major platelet receptors (GPIb-IX-V, GPVI, and αIIbβ3) [6,11].

Since PI3K inhibitors represent favorable candidates for the prevention of thrombosis, several pharmacological approaches have been carried out in order to elucidate the role of PI3K isoforms in platelet function [6]. PI3Kβ has been revealed as the main Class I isoform in platelet function and thrombosis [9,12]. The pharmacological effects of selective p110β inhibitors such as TGX-221 and AZD6482 both in preclinical and clinical settings are important to highlight the role of PI3Kβ in platelet activation and thrombus formation [8,13]. Indeed, the deletion of p110β in megakaryocytes/platelets leads to a reduced platelet activation downstream of the collagen receptor GPVI and the integrin αIIbβ3, and also an impaired in vivo thrombosis following FeCl3 injury in mice [12,14]. However, p110β-deficient mice show enhanced embolization both ex vivo and in vivo at higher shear rates in the formed thrombi. 

The present study focused on a promising antiplatelet drug, Idelalisib, which targets the δ catalytic subunit of PI3K, which has been poorly investigated in this context. This isoform is almost specific to hematopoietic cells and seems to play a minor role in platelet activation. The first-in-class oral selective inhibitor of p110δ isoform Idelalisib has been approved for the treatment of hematological malignancies including chronic lymphocytic leukemia (CLL), follicular B-cell non-Hodgkin lymphoma (FL), and small lymphocytic lymphoma (SLL) [15,16], and has shown considerable antitumor activity in the treatment of hematological cancer [17]. In this respect, Idelalisib is currently combined with rituximab as a second-line agent in intractable patients or patients with other co-morbidities [18]. After oral administration, the median absorption time is 1.5 h whereas half-life of the compound is 8.2 h [16,18]. Limited data on Idelalisib-associated bleeding tendencies have been reported to date [19]. However, Reda et al. observed that Idelalisib-treated CLL patients do not show hemorrhagic complications or exacerbate bleeding and might ameliorate platelet function [20]. A potential explanation to this discrepancy might be related to the inherent differences between human and mouse platelets, including the absence on mouse platelets of important surface receptors, such as the IgG receptor, FCγRIIA, and the thrombin receptor, PAR1 [21,22].

Consistent with the previous known role of PI3K in GPVI signalling [23], we report that Idelalisib showed antiplatelet activity specially when PRP and washed platelets were stimulated with suboptimal concentrations of CRP, in comparison with stimulation with collagen and rhodocytin. These results support previous studies on p110δ knock-out mice demonstrating that p110δ plays a certain role in mediating platelet activation by GPVI [11]. In clinical trials, platelet aggregation induced by collagen was not affected during the study period in Rituximab-Idelalisib-treated CLL patients [20], although hemodynamic function regarding platelet aggregation might differ from ex vivo experiments, as platelets isolated from blood can elicit different responses. Nevertheless, the present data elucidated that Idelalisib appears to be an effective modulator of CRP-induced aggregation pointing towards the PI3K pathway as a potential player in GPVI signalling. In line with the above results, adhesion experiments demonstrated that Idelalisib reduced platelet adhesion on immobilized type I collagen, as well as adhesion and aggregation under high shear rates, reinforcing the role of PI3Kδ in GPVI signalling. In this context, GPVI, the major collagen receptor, which is only expressed on platelets and megakaryocytes [24,25], is a promising antithrombotic target for which no drugs are available. GPVI inhibition has demonstrated antithrombotic efficacy in experimental models of thrombosis without enhancing bleeding [26,27]. Further validation of GPVI as a therapeutic target would be of potential interest for introducing innovations based on ideal attributes such as minimizing bleeding for the treatment of certain thrombosis [28].

Thrombosis studies in mice demonstrated that Idelalisib treatment resulted in decreased arterial thrombosis without affecting the bleeding time at the same level than other antiplatelet drugs. Bleeding is a major complication of common antiplatelet therapy in humans and a concern when developing new antiplatelet therapies [29]. The present data show that intracellular PI3Kδ inhibition significantly increased the bleeding time compared to DMSO-treated mice, but this increment is far less pronounced than that with aspirin or clopidogrel. This highlights that Idelalisib is a favorable antithrombotic drug in terms of minimizing the bleeding risk. Indeed, Idelalisib-treated mice had extremely reduced in vivo thrombus formation showing equal occlusion time as clopidogrel [30].

Collectively, our results suggest that selective PI3Kδ inhibition with Idelalisib prevents platelet activation by ITAM-related signalling pathways, especially GPVI. We also show that this inhibition leads to an abrogation of platelet adhesion to collagen and a thrombus formation reduction in mice associated with minor bleeding. Therefore, our study indicates Idelalisib may have antiplatelet effects and provides a rationale to evaluate its antithrombotic therapeutic efficacy in humans.

## 4. Materials and Methods

### 4.1. Human Blood Collection and Platelet Isolation

Fresh blood was obtained from healthy volunteers who were not under chronic medication, or under antiplatelet drugs for the previous 10 days. Blood samples were collected in coagulation 3.2% sodium citrate tubes (Vacuette^®^, Greiner Bio-One International GmbH, Kremsmünster, Austria), and processed in less than 30 min after the extraction.

For platelet isolation, a robust procedure designed to limit other contamination from blood was followed [31]. After washing steps, platelets were resuspended in HEPES-Tyrode’s (134 mM NaCl; 0.34 mM Na2HPO4; 2.9 mM KCl; 12 mM NaHCO3; 20 mM HEPES; 5 mM glucose; 1 mM MgCl2; pH 7.3) at the desired concentration followed by a resting step of 30 min at room temperature. 

### 4.2. Intracellular Ca^+2^ Mobilization Assays

Washed platelets (5 × 10^6^) were incubated with FLIPR Calcium 4 reagent (Molecular Devices LLC, San Jose, CA, USA) in a 384-well transparent bottom plate (Greiner Bio-One International GmbH, Kremsmünster, Austria) and supplemented with Probenecid (Invitrogen™, Carlsbad, CA, USA) 0.07% for 1 h at 37 °C. Platelets were co-incubated for 10 min with the PI3K inhibitor (10 μM) and inhibitors of secondary mediators (2 U/mL apyrase and 10 μM indomethacin (Sigma-Aldrich, Saint Louis, MO, USA)) under non-aggregating conditions (9 μM eptifibatide (Cayman Chemicals, Ann Arbor, MI, USA)). Then, the GPVI-specific agonist Collagen-Related Peptide (CRP) was added to the platelet suspension at different doses. Both PI3K inhibitors and CRP were automatically dispensed by FDSS7000EX Functional Drug Screening System (Hamamatsu Photonics, Hamamatsu, Japan). Intracellular Ca^2+^ mobilization was assessed by measuring fluorescence changes (480 nm emission and 540 nm excitation wavelengths) in real time for 15 min after the addition of the agonists (Figure 1A). 100% was expressed as maximal Ca^2+^ release in platelets treated with the agonist CRP. To determine IC_50_, compounds were tested at 9 different concentrations in duplicates starting from 10 μM and performing 1:3 serial dilutions. The analysis of the data was performed with GraphPad Prism^®^ software (version 6.0e, March 2014) (GraphPad Software, San Diego, CA, USA) by carrying out a nonlinear regression fit for log(inhibitor) vs. relative CRP activity using a Hill slope of −1, and top and bottom constraints of 100 and 0, respectively.

### 4.3. Platelet Aggregation-Based Selectivity Assays

Aggregations were performed in platelet-rich-plasma (PRP) or washed platelets previously warmed at 37 °C for 4 min without stirring and for 1 min with constant stirring at 1200 rpm in a Chrono-log^®^ 490-X aggregometer (Chrono-log Corporation, Havertown, PA, USA). After that, PRP or washed platelets were stimulated with the selected agonists for 5 min. The agonists used in the assay were: CRP-XL (Cambcol Laboratories Ltd., Cambridgeshire, UK), Horm^®^ collagen (Takeda Austria GmbH, Linz, Austria), rhodocytin (provided by Johannes A. Eble, from the Institute of Physiological Chemistry and Pathobiochemistry, University of Münster, Münster, Germany), thrombin receptor activating peptide (TRAP) (Tocris Bioscience, Bristol, UK), and thrombin (Sigma-Aldrich, Saint Louis, MO, USA).

### 4.4. Viability-Related Calcein-AM Flow Cytometry Assay

To measure the potential toxicity of inhibitors, a viability assay based on Calcein-AM binding measured by flow cytometry was performed. Washed platelets (2.5 × 10^8^ platelets/mL) were incubated for 10 min with 10 μM Idelalisib (MedChemExpress, Shanghai, China) or 0.1% DMSO (Sigma-Aldrich, Saint Louis, MO, USA) as vehicle. Then, 1 μL of Calcein-AM 0.1 mg/mL (Sigma-Aldrich, Saint Louis, MO, USA) was added to the suspension and incubated for an additional 20 min at room temperature. Calcein-AM binding was measured in triplicate by an Accuri C6 flow cytometer (BD Biosciences, San Jose, CA, USA). Data were analyzed comparing the average of the percentage of positive events in the presence of the inhibitor with the average of the percentage of positive events in the absence of the inhibitor (vehicle). 

### 4.5. Platelet Static Adhesion Assays

Washed platelets (1 × 10^8^ platelet/mL) were added to 96-well assay plates (Nunc MaxiSorpTM Thermo Fisher Scientific, Waltham, MA, USA) previously coated overnight with 5 μg/mL Horm® collagen (Takeda Austria GmbH, Linz, Austria), and incubated for 1 h at 37 °C. Unbound platelets were separated by washing the plates with PBS three times. Then, *p*-nitrophenyl phosphate-based solution was added to plates for 40 min with agitation. Reactions were stopped with 50 μL of 3 M NaOH and the absorbance (405 nm) was measured in a Tecan Infinite^®^ M1000 plate reader (Tecan Group Ltd., Männedor, Switzerland). Each assay was run three times and absorbance values (arbitrary units) were normalized with the negative control (BSA condition).

### 4.6. Evaluation of Platelet Adhesion and Aggregation by the Impact-R Test 

Venous blood was obtained from five healthy volunteers, into buffered 3.2% sodium citrate. Blood samples were incubated with either vehicle (DMSO 0.1%) or Idelalisib 10 μM (10 min at RT) an immediately assayed on the Impact-R^TM^ cone and plate analyzer (Matis Medical Inc.-DANED SA, Beersel, Belgium), as previously described. [32] Briefly, 130 μL of blood mixture were placed, in duplicate, into a polystyrene well. After that, a Teflon cone was placed on top and rotated (2050/s) to produce a shear rate (1800 s^−1^) leading to platelet adhesion and aggregation to the plastic. After washing the well and staining with May-Grünwal solution, the plate surface covered with stained objects (%SC), indicating platelet adhesion, and the average size of the objects AS (μm^2^), indicating platelet aggregation, were quantified using the inverted light microscope connected to a camera and an image analyzing software. Median values were calculated from 7 images taken per well.

### 4.7. Animal Studies

C57BL/6J mice (Envigo Rms Spain Sl., Sant Feliu de Codines, Spain) were kept under controlled environmental conditions (relative humidity: 45–65%; temperature: 20–24 °C) with a 12 h light/dark cycle and free access to chow and water. At the time of the study, mice were 8–12 weeks old. Equivalent numbers of males and females were included. According to the oral drug administration protocol, mice were immobilized and Idelalisib, 20 mg/kg or vehicle (DMSO; Sigma-Aldrich, Madrid, Spain), was introduced directly into the stomach with an oral gavage feeding tube. One hour after the treatment, tail bleeding and arterial thrombosis assays were performed. A separate set of animals treated with other established antiplatelet drugs were also used as positive controls. Acetyl salicylic acid (ASA; Sigma-Aldrich, Madrid, Spain) was dissolved in water at 1 mg/mL and given by a single intraperitoneally injection of 100 mg/kg in mice 2–4 h before the experiment, as previously reported [33]. Moreover, two doses of clopidogrel (2 mg/kg) were also given to both animal groups by oral gavage 24 h and 2 h before the experiment.

### 4.8. Tail-Bleeding Time

After anesthetizing mice with isoflurane, tails were amputated 3 mm from the tip and submerged into saline solution at 37 °C. Bleeding time was recorded until the bleeding stopped (no further bleeding within 1 min) or for 10 min at maximum. A quantitative estimation of the amount of blood was determined by measuring the hemoglobin (Hb) content of blood collected in saline. For this purpose, tubes were centrifuged and erythrocyte lysis buffer (Qiagen, Madrid, Spain) was added to samples. Absorbance was assessed at 575 nm in a microplate reader (Biotek^®^ Synergy, Winooski, VT, USA). Bleeding times were performed blind to the mice treatment.

### 4.9. Chemically Induced Arterial Thrombosis In Vivo

Animals were anesthetized by intraperitoneal injection (xylazine hydrochloride 10 mg/kg + ketamine hydrochloride 10 mg/kg). Thrombus formation was induced by applying a piece of filter paper (5 × 1 mm) soaked with a 7.5% ferric chloride solution (FeCl_3_, Sigma-Aldrich, Madrid, Spain) to the left carotid artery for 2 min. A Doppler ultrasound flow probe (Model 0.5 PSB, Transonic Systems) and a flow meter (Model TS420, Transonic Systems, Ithaca, NY, USA) were used to continuously register the blood flow. Occlusion time was specified as the time elapsed from the withdrawal of FeCl_3_ to the lack of blood flow (≤0.01 mL/min) for at least 3 consecutive minutes. Experiments were stopped after 30 min if no occlusion occurred. 

### 4.10. Analysis of Idelalisib in Mice Plasma

Whole blood from mice was collected after 1 h-Idealisib treatment by retro-orbital bleeding in tubes with citrate. Platelet-poor plasma (PPP) was isolated by centrifugation of whole blood (1500 g for 5 min at RT). Idelalisib (purity 99.98%) was purchased from MedChemExpress (Shanghai, China). Formic acid, methanol and acetonitrile were purchased from Scharlab (Barcelona, Spain). Idelalisib at 10 mM stock solution was prepared in acetonitrile. This stock solution was diluted in 20% acetonitrile at the concentrations of 1000, 500, 250, 125, 62.5, 31.2, 15.6, and 7.8 μM. The calibration curve was constructed by the analysis of mouse plasma samples spiked with the mentioned varying concentrations of Idelalisib. For sample preparation prior to LC–MS/MS, a liquid extraction was carried out. Briefly, individual aliquots of 50 μL of plasma samples were dissolved 1:1 in acetonitrile–water solution (*v*/*v*, 1:9) and centrifuged at 2235 g for 60 min. Then, 4 μL of the clean supernatant was injected into the LC–MS/MS system. Waters Acquity tandem quadrupole mass spectrometer Xevo TQD (Waters, Milford, MA, USA) was used for the analysis. For HPLC separation, Waters Acquity UPLC BEH C18 column was subjected to a flow rate of 0.6 mL/min by using a mobile phase consisting in 0.1% formic acid in water (Solvent A) and acetonitrile (Solvent B) in the following gradient elution: 0–0.1 min, 5% B; 0.1–1 min, 5% B, 1–2 min, 5–100% B; 2–2.1 min, 5% B; and 2.1–3.0 min, 5% B. The software used for instrument control and data analysis was MassLynx™ version 4.1 SCN 940 (Waters, USA). The MS/MS detection was conducted operating in positive ion mode by monitoring the fragmentation of m/z 416.11 → 176.1 m/z. The calibration curve of Idelalisib was constructed by plotting the peak area ratio (PAR) in relation to each known concentration of the drug. The slope and correlation coefficient (r^2^ = 0.999493) were determined from the known concentrations of the drug by linear regression analysis. The unknown drug concentrations were determined by extrapolating PAR values from each sample to the calibration curve.

### 4.11. Statistical Analysis

Normality of data was checked using the Shapiro-Wilk normality test. Non-parametric tests were used when the data did not pass the normality tests. Differences between groups were analyzed by paired or unpaired *t*-test as well as ANOVA as appropriate. *p* values of <0.05 were considered significant.

## Data Availability

The data presented in this study are available in this article and the supplementary material.

## Supplementary Materials

The following are available online at https://www.mdpi.com/1422-0067/22/7/3304/s1.
