# Supplementary material for: The PI3Kδ Inhibitor Idelalisib Diminishes Platelet Function and Shows Antithrombotic Potential
Source: Int J Mol Sci. 2021 Mar 24;22(7):3304. doi: 10.3390/ijms22073304 (PMC8037016; doi:10.3390/ijms22073304)
Supplement: Supplementary file 1 [file ijms-22-03304-s001.pdf]

SUPPLEMENTARY MATERIALS

The PI3Kδ Inhibitor Idelalisib Diminishes Platelet Function and Shows Antithrombotic Potential

María N. Barrachina [1,†] Irene Izquierdo [1,†], Lidia Hermida-Nogueira [1], Luis A. Morán [1], Amparo Pérez [2,3], Ana B. Arroyo [4], Nuria García-Barberá [4], Rocío González-Conejero [4], Sara Troitiño [1], Johannes A. Eble [5], José Rivera [4], Constantino Martínez [4], María I. Loza [2,3], Eduardo Domínguez [2,3,#] and Ángel García [1, #,*]

[1] Platelet Proteomics Group, Center for Research in Molecular Medicine and Chronic Diseases, Universidade Santiago de Compostela and Instituto de Investigación Sanitaria de Santiago, 15706 Santiago de Compostela, Spain; Maria.Barrachina@childrens.harvard.edu (M.N.B.); irene.izquierdo.b@gmail.com (I.I.); lidia.nogueira@usc.es (L.H.-N.); luisarturo.moran@usc.es (L.A.M.); sara.troitino.cora@rai.usc.es (S.T.)

[2] Pharmacology Applied to Drug Discovery group, Centro Singular de Investigación en Medicina Molecular y Enfermedades Crónicas, Universidade Santiago de Compostela, 15705 Santiago de Compostela, Spain; amparo.perez@usc.es (A.P.); mabel.loza@usc.es (M.I.L.); eduardo.dominguez@usc.es (E.D.)

[3] Grupo Biofarma, Instituto de Investigación Sanitaria de Santiago, 15706 Santiago de Compostela, Spain.

[4] Servicio de Hematología y Oncología Médica, Hospital Universitario Morales Meseguer, Centro Regional de Hemodonación, Universidad de Murcia, IMIB-Arrixaca, CIBERER-U765, 30003 Murcia, Spain; anabelen.arroyo@um.es (A.B.A.); nurgarbar@gmail.com (N.G.-B.); rocio.gonzalez@carm.es (R.G.-C.); jose.rivera@carm.es (J.R.); constant@um.es (C.M.)

[5] Institute of Physiological Chemistry and Pathobiochemistry, University of Münster, 48149 Münster, Germany; johannes.eble@uni-muenster.de (J.A.E)

* Correspondence: angel.garcia@usc.es; Tel.: +34 881 815429

† These two authors contributed equally to the work; # These two authors contributed equally to the work.

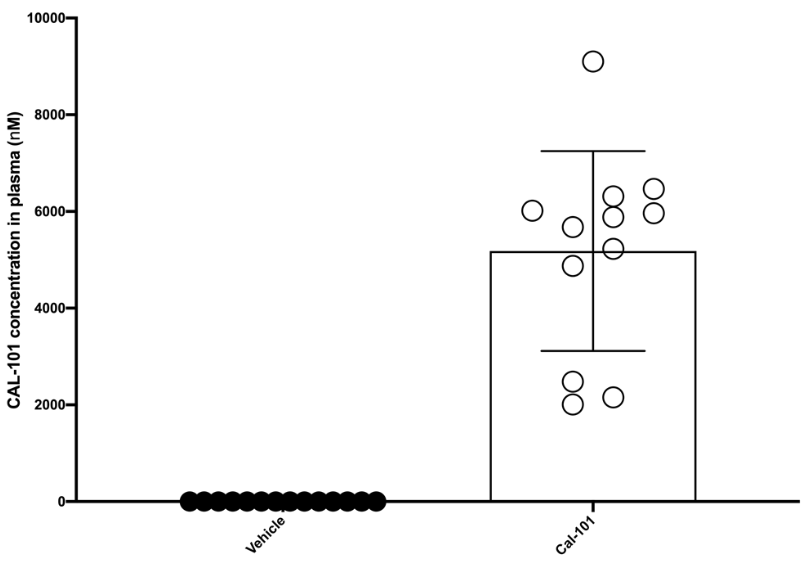

Supplementary Figure S1. Idelalisib (Cal-101) plasma concentration in mice undertaking 2 mg/kg of the drug.

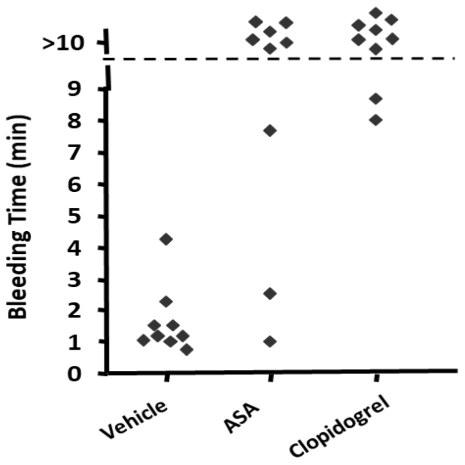

Supplementary Figure S2. Tail bleeding times for mice treated with vehicle (controls), acetyl salicylic acid (ASA), and clopidogrel.
